# Emotional Responses and Perceived Relative Harm Mediate the Effect of Exposure to Misinformation about E-Cigarettes on Twitter and Intention to Purchase E-Cigarettes among Adult Smokers

**DOI:** 10.3390/ijerph182312347

**Published:** 2021-11-24

**Authors:** Jessica Liu, Caroline Wright, Olga Elizarova, Jennifer Dahne, Jiang Bian, Andy S. L. Tan

**Affiliations:** 1 Department of Social and Behavioral Sciences, Harvard TH Chan School of Public Health, Harvard University, Boston, MA 02115, USA; 2 Department of Population Health Sciences, Bristol Medical School, University of Bristol, Bristol BS8 2BN, UK; caroline.wright@bristol.ac.uk; 3 Play Collaborate Change, Boston, MA 02128, USA; olga_elizarova@alumni.brown.edu; 4 Department of Psychiatry and Behavioral Sciences, College of Medicine, Medical University of South Carolina, Charleston, SC 29425, USA; dahne@musc.edu; 5 Hollings Cancer Center, Medical University of South Carolina, Charleston, SC 29425, USA; 6 Health Outcomes & Biomedical Informatics, College of Medicine, University of Florida, Gainesville, FL 32610, USA; bianjiang@ufl.edu; 7 Annenberg School for Communication, University of Pennsylvania, Philadelphia, PA 19104, USA; andy.tan@asc.upenn.edu; 8 Leonard Davis Institute of Health Economics, University of Pennsylvania, Philadelphia, PA 19104, USA

**Keywords:** social media, misinformation, Twitter, e-cigarettes, emotions

## Abstract

There is a gap in knowledge on the affective mechanisms underlying effects of exposure to health misinformation. This study aimed to understand whether discrete emotional responses and perceived relative harm of e-cigarettes versus smoking mediate the effect of exposure to tweets about the harms of e-cigarettes on Twitter and intention to purchase e-cigarettes among adult smokers. We conducted a web-based experiment in November 2019 among 2400 adult smokers who were randomly assigned to view one of four conditions of tweets containing different levels of misinformation. We fitted mediation models using structural equation modeling and bootstrap procedures to assess the indirect effects of exposure to tweets through perceived relative harm of e-cigarettes and six discrete emotions. Our findings support that exposure to tweets about harms of e-cigarettes influence intention to purchase e-cigarettes through perceived relative harm, discrete emotional responses, and serially through emotional responses and perceived relative harm. Feeling worried, hopeful, and happy mediated the effects of condition on intention to purchase e-cigarettes. Feeling scared, worried, angry, and hopeful mediated the effects serially through perceived relative harm. Affective responses and perceived relative harm following exposure to misinformation about e-cigarette harm may mediate the relationship with intention to purchase e-cigarettes among adult smokers.

## 1. Introduction

There is increasing evidence of the proliferation of online misinformation, defined as information that is factually incorrect or misleading [1], with regards to e-cigarettes on social media including Twitter [2,3]. Misinformation about tobacco products is not a new phenomenon as the tobacco industry has perpetuated falsehoods to downplay and deny harms and addictiveness of combustible cigarettes over decades. However, with the introduction of alternative forms of nicotine delivery such as e-cigarettes, the information landscape surrounding e-cigarette use and relative harms against cigarette smoking has become more complex. For instance, based on the current state of research around e-cigarette harms [4,5], misinformation related to e-cigarette harms may include statements that downplay the risks and addictiveness of e-cigarettes [6]. Conversely, misinformation about e-cigarette harms may exaggerate the harms of e-cigarettes such as through claims that they are just as or more harmful than smoking combustible cigarettes. In the context of social media, a rising platform for consuming health information, both types of misinformation may be amplified more widely and can influence decisions about the uptake of e-cigarettes versus continuation of smoking combustible cigarettes. 

Current evidence suggests that the short-term health risks of using e-cigarettes are lower than smoking combustible cigarettes [4,5]. Smokers who quit smoking combustible cigarettes and switch to e-cigarettes completely may reduce their risk and exposure to cigarette smoke and smoking-related illnesses [5,7]. Yet, recent studies among current smokers reported misperceptions about the relative harm and risks of e-cigarettes are increasing in both the United Kingdom (UK) and the United States (US) [8]. Fewer adult smokers in the UK believed that e-cigarettes were less harmful than cigarettes in 2019 (34%) compared with smokers in 2014 (45%) [5]. Similar trends were observed among smokers in the US where 26% of smokers believed that e-cigarettes were less harmful than smoking cigarettes in 2018 compared with 29% of smokers in 2017 [9]. Conversely, more smokers believed e-cigarettes were much more harmful than smoking cigarettes in 2018 (4%) than in 2017 (2%) [5]. 

Misperceptions about the relative harm of e-cigarettes compared with smoking may deter smokers from using e-cigarettes to help with quitting smoking [10,11]. However, we also highlight that e-cigarette use is not completely harmless. Using e-cigarettes is associated with potential health risks due to inhalation of particulate matter, detectable levels of potential carcinogens, and other constituents with known health risks [4]. Nevertheless, the US Centers for Disease Control and Prevention (CDC) acknowledges that smokers can potentially benefit from e-cigarettes if they used them as a complete substitute for combustible cigarettes [12]. 

To better understand the impact of misinformation on social media on population health, Chou and colleagues called for five key areas of research that are currently understudied, one of which included understanding the psychological drivers of misinformation acceptance and sharing [13]. There is currently a research gap in understanding the roles of emotion and cognition in relation to e-cigarette misinformation on behavioral outcomes in the adult population who are smoking cigarettes. We found one study, in the context of infectious disease outbreaks, that reported social media use can significantly increase preventive behaviors via fear and anger and the public’s risk perception [14]. Other studies examined the diffusion of emotional content on social media, such as how moral-emotional messaging on social media tends to spread more within ideological groups than between groups [15]. Studies have also analyzed the prevalence of emotions in Facebook posts, with one study finding that positive emotions are more prevalent than negative emotions when browsing Facebook [16], and another study explored emotional contagion [17]. There is a need for more evidence of the emotional and cognitive drivers of the influence of misinformation on health behaviors [18].

To address this research gap, we conducted a web-based randomized controlled experiment to assess the effect of exposure to misinformation in the specific context of social media content about e-cigarette harm on Twitter [19]. We aimed to analyze whether emotions and perceived relative harms of e-cigarettes, elicited by exposure to e-cigarette misinformation, influenced adult smokers’ intention to purchase e-cigarettes. In a previously published paper using these data, we found that condition (either viewing tweets that e-cigarettes were just as or more harmful than smoking, e-cigarettes were completely harmless, e-cigarette harms were uncertain, or control tweets about physical activity) was associated with intention to purchase e-cigarettes and perceived relative harm perceptions and was not associated with quit intention [20]. 

We test the hypotheses that emotions and perceived relative harm mediate the influence of exposure to misinformation on social media on smokers’ intention to purchase e-cigarettes. 

We will be exploring this mediation pathway informed by the Appraisal Tendency Framework [21]. The Appraisal Tendency Framework provides a nuanced explanation of discrete emotions in shaping perceptions and behavioral outcomes, with each emotion distinctively leading to differential risk perceptions and behavioral consequences (Lerner and Keltner, 2001). Based on previous findings and theoretical frameworks, distinct emotions and perceived risk are expected to be the psychological mechanism through which exposure to misinformation on social media influences e-cigarette related behaviors, such as intention to purchase [14].

**Figure 1:**
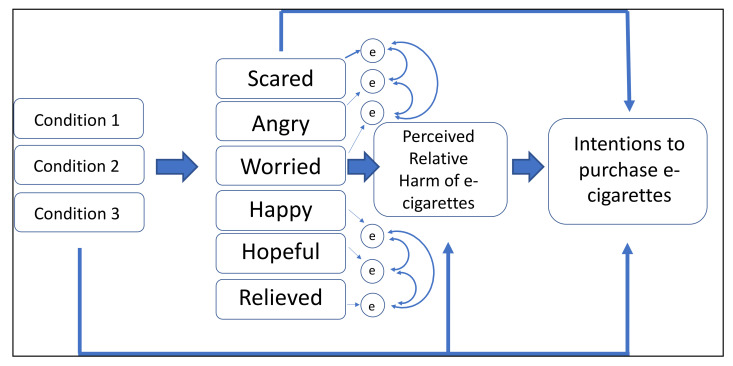
Hypothesized Structural Equation Model, informed by the Appraisal Tendency Framework. Note. The model adjusted for baseline perceived relative harm and intentions to purchase e-cigarettes.

Specifically, we propose the following hypotheses to examine how exposure to one of four types of information affects perceived relative harm and intention to purchase e-cigarettes through six distinct emotions: scared, angry, worried, happy, hopeful, and relieved. The four types of exposure to information, or conditions, were: E-cigarettes are as or more harmful than smoking (Condition 1), E-cigarettes are completely harmless (Condition 2), Uncertainty of e-cigarette harm (Condition 3), or information on physical activity (Condition 4). Conditions 1 and 2 represent misinformation about e-cigarette harms. Condition 3 represents tweets that highlight how e-cigarette harms are not yet known. Condition 4 represents the control condition. A summary of our hypotheses is depicted in Figure 1.

**Hypothesis 1** **(H1).** *Conditions 1, 2, and 3 (vs. Condition 4) will have an indirect effect on intention to purchase e-cigarettes through perceived relative harm of e-cigarettes*.

**Hypothesis 2** **(H2).** *Conditions 1, 2, and 3 (vs. Condition 4) will have an indirect effect on intention to purchase e-cigarettes through each emotion*.

We also propose a serial mediation model.

**Hypothesis 3** **(H3).** *Conditions 1, 2, and 3 (vs. Condition 4) will be associated with intention to purchase e-cigarettes through each emotion and perceived relative harm of e-cigarettes in serial*.

Findings from this study can provide new insights on the psychological and emotional drivers of misinformation exposure through social media on behaviors and inform efforts to develop appropriate interventions to mitigate these effects [13].

## 2. Methods

### 2.1. Sampling and Recruitment 

**Figure 2:**
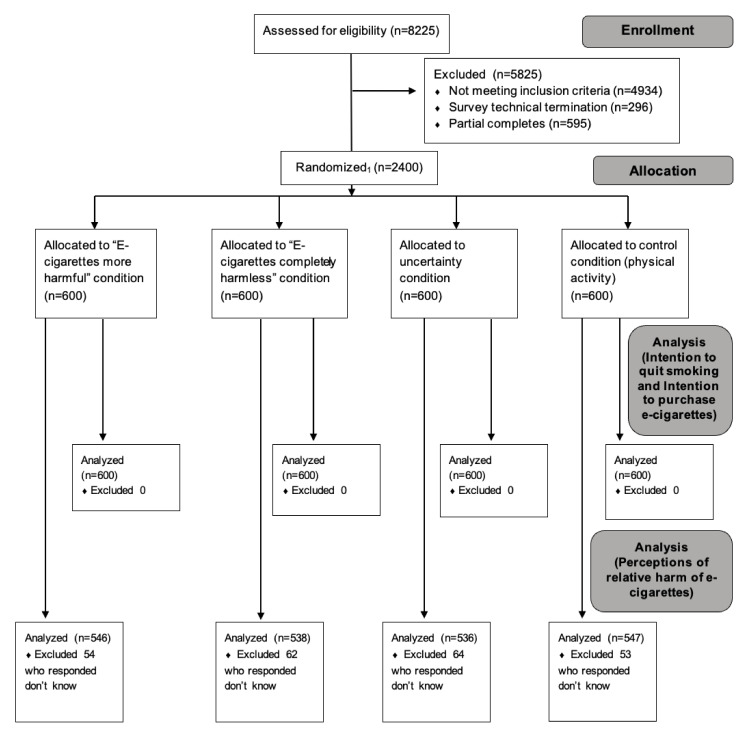
CONSORT Flow Diagram. 1. Survey recruitment used a least-fill approach; as a respondent came in, they were assigned to the exposure with the lowest complete count.

Eligible participants were adult smokers aged 18 years and older who reported cigarette use and no e-cigarette use in the past 30 days. We enrolled 2400 participants from the US and UK through the Prodege consumer research panel who were recruited through email, telephone, messaging on websites, and online communities, and received reward points after completing the study (see Figure 2: CONSORT diagram). Participants were consented using a form on the survey platform. The University of Bristol’s Institutional Review Board approved this study.

### 2.2. Twitter Message Stimuli Selection

Details of the message stimuli selection from Twitter are described elsewhere [20]. Briefly, we conducted a keyword search of 1% of tweets relevant to e-cigarette harms on Twitter between January and September 2019 (n = 499 tweets). The sample of tweets was reduced to approximately 20 per condition. We reached consensus on whether each tweet would be suitable as experimental stimuli based on the following: (1) explicit messaging that e-cigarettes were either more harmful than smoking, completely harmless, or uncertain, (2) no mention of youth, (3) no mention of diseases, (4) no profanity, (5) had ‘likes’ or ‘retweets’, (6) no advertising, (7) no pictures, (8) and was publicly available deleted. Next, we chose four tweets for the three conditions: (1) E-cigarettes are as or more harmful than smoking, (2) E-cigarettes are completely harmless, and (3) Uncertainty. It is important to understand the effects of exposure to Tweets that include uncertainty of e-cigarette harms, as media and public discourse around this topic have included uncertainty as evidence-based information around e-cigarette harms have been developing [20]. The control condition comprised four tweets about physical activity. We selected physical activity messages as the control to reduce bias due to experimenter demand. The tweets for each condition can be found in the Supplementary Materials. 

### 2.3. Study Procedure and Data Collection

Eligible participants who consented were first asked to complete baseline measures of intention to quit smoking, intention to purchase e-cigarettes, and beliefs of e-cigarette harms. They were then randomized to view four tweets within each of the Conditions: (1) E-cigarettes are as or more harmful than smoking, (2) E-cigarettes are completely harmless, (3) Uncertainty, or (4) Control (physical activity). Participants were randomized to these conditions in a 1:1:1:1 ratio using the randomizer function on the Prodege survey platform. After randomization to a condition, participants saw one tweet at a time in random order and were asked questions on the perceived effectiveness and likelihood of engagement with the tweet. They next completed post-test measures of emotional responses and behavioral intention [20], beliefs of e-cigarette harms, their tobacco use behaviors, media exposure and use, and sociodemographic characteristics. 

### 2.4. Measures

Baseline and post-test intention to purchase e-cigarettes were measured using the following question adapted from the Juster Scale: [22] “How probable is it that you will purchase e-cigarettes/vapes in the next month?” Response options ranged from 0 (No chance, almost no chance) to 10 (Certain, practically certain). 

Baseline and post-test perceived relative harm of e-cigarettes versus smoking were measured using the following question used in prior surveys: [9,23] “Compared to smoking regular cigarettes, would you say that e-cigarettes/vapes are…” Response options ranged from 1 (Much less harmful) to 5 (Much more harmful) and included a “Don’t know” option. Responses indicating “Don’t know” were recoded as missing values and not included in the analysis (177 missing baseline, 169 missing post-test, and 233 missing either baseline or post-test values). 

Emotional responses to viewing the tweets were measured using the following question: “When thinking about e-cigarettes/vapes, do the messages you just saw make you feel… Scared/Hopeful/Worried/Happy/Angry/Relieved?” Response options for each emotion ranged from 1 (Not at all) to 5 (Completely).

Demographic characteristics of participants were measured through questions about age, sex, race/ethnicity, education, cigarette use, social media use, and daily internet use. 

### 2.5. Data Analysis

We fitted a structural equation model using Stata version 14.0 (College Station, TX, USA) to examine how exposure to one of four types of Tweets affects perceived relative harm and intention to purchase e-cigarettes directly and indirectly through six distinct emotions, as depicted in our hypothesized model in Figure 1. The model controlled for baseline intention to purchase e-cigarettes and baseline perceived relative harm of e-cigarettes. The control condition (physical activity promotion) was the comparison condition. An initial model with no correlated errors between emotions had poor fit with the data. Based on the model fit and modification indices, we fitted a second model that added correlated errors among the positive emotions and correlated errors among the negative emotions and this improved the fit significantly and retained this as our final model (RMSEA = 0.066, CFI = 0.992, TLI = 0.949, SRMR = 0.026). To address the three mediation hypotheses (through relative harm (H1), emotions (H2), and serial mediation through emotions and relative harm (H3)), we ran the mediation model with a bootstrapping procedure (with 1000 replications). We used the bootstrap approach because this method has high power and does not rely on the assumption that estimates of the indirect effects are normally distributed [24]. We computed the indirect effect coefficients as a product of the coefficients for condition x relative harm (H1), condition x each emotion (H2), and condition x each emotion x relative harm (H3). The bootstrap procedure was used to compute the bias-corrected confidence intervals and we assessed whether each indirect effect was statistically significant by observing if the confidence interval included zero. Listwise deletion was used to deal with values missing on baseline and/or post-test perceived relative harm of e-cigarettes. 

## 3. Results

### 3.1. Demographics

Table 1 summarizes the sample demographics by experimental condition and country. In the overall sample, participants ranged from 18–84 years of age (mean = 47.0, SD = 14.6), and 5.8% were between the ages of 18–24 and 94% were ages 25 and older. 47% were female and 53% were male in the overall sample. A majority of the cohort (82%) smoked cigarettes daily and over half (52%) reported lifetime use of e-cigarettes. For the US participants, 71% were White, 17% were Black/African American, and 12% identified as other racial groups. Among the UK participants, 93% were White and 7% were from other ethnic backgrounds. The total number of social media types used more than once per day by participants was a mean of 2.0 (SD of 1.6), and the mean daily internet use of the participant sample was 16.3 (SD = 7.0) hours per day. Our analysis showed that randomization had been successful. 

### 3.2. Descriptive Analysis of Key Variables and Bivariate Correlations

Table 2 summarizes the descriptive analyses of the key variable in the mediation model including intention to purchase e-cigarettes, perceived relative harm, and emotional responses and shows their zero-order correlations with experimental condition. Table 3 summarizes each emotion by condition, the mean and standard deviation for: (1) each discrete emotion, (2) baseline and post-test perceived relative harm of e-cigarettes, and (3) baseline and post-test intentions to purchase e-cigarettes, for the entire sample as well as stratified by US and UK.

### 3.3. Testing Hypothesis 1

H1 predicted that the effect of condition on intention to purchase e-cigarettes will be mediated through perceived relative harm. H1 was partly supported: Two of three mediation paths through relative harm were significant (Condition 1 and 2 vs. Condition 4) (Table 4). Those in Condition 1 had lower intention to purchase e-cigarettes because of increased perceived relative harm while those in Condition 2 had higher intention to purchase e-cigarettes because of lower relative harm. The mediation path through relative harm comparing Condition 3 and 4 was not significant.

### 3.4. Testing Hypothesis 2

H2 predicted that the effect of condition on intention to purchase e-cigarettes will be mediated through each discrete emotion. H2 was partly supported: Eight of the 18 mediation paths through emotions are significant (through Worried, Hopeful, and Happy) (Table 4). Specifically, comparing Condition 1 vs. Condition 4, Worried, Hopeful, and Happy were significant mediators; comparing Condition 2 vs. Condition 4, Hopeful and Happy were significant mediators; and comparing Condition 3 vs. Condition 4, Worried, Hopeful, and Happy were significant mediators.

### 3.5. Testing Hypothesis 3

H3 predicted that the effect of condition on intention to purchase e-cigarettes will be serially mediated through each discrete emotion and perceived relative harm. H3 was partly supported: Nine of the 18 serial mediation paths through emotions and perceived relative harm are significant (through Scared, Worried, Angry, and Hopeful) (Table 4). Specifically, comparing Condition 1 vs. Condition 4, Scared, Angry, and Hopeful were significant mediators; comparing Condition 2 vs. Condition 4, Angry and Hopeful were significant mediators; and comparing Condition 3 vs. Condition 4, Scared, Worried, Angry, and Hopeful were significant mediators.

### 3.6. Differences between US and UK

We did not find differences between the US and UK in terms of mediation effects. We performed invariance testing, corrected for multiple comparisons, and reviewed model fits, and ultimately decided to retain the original model because of no improvement in model fit after allowing parameters to vary between the US and UK.

## 4. Discussion

Our analysis provided partial support of hypotheses that misinformation effects are mediated through perceived relative harm of e-cigarettes, discrete emotions, and serially through emotions and perceived relative harm. Three of the six discrete emotions mediated the effects of condition on intention to purchase e-cigarettes (worried, hopeful, and happy) while 4 discrete emotions mediated the effects serially through perceived relative harm (scared, worried, angry, and hopeful). Individually, the mediated effects of condition through each emotion and the serial mediation effects were small. However, the cumulative mediated effects through emotions may explain a meaningful proportion of the effects of condition on the intention to purchase e-cigarettes. 

Chou and colleagues described that understanding the psycho-socio-emotional drivers of misinformation acceptance is an important first step for developing successful interventions [13]. Simply providing factual information to counter misinformation may prove ineffective if we do not also address the emotions associated with encountering misinformation. Our study findings address a key gap in the literature to help inform the design of successful interventions to disrupt the negative impacts of health misinformation on social media through emotional and cognitive pathways. We learnt that certain emotions and not all of the six emotional responses we evaluated were significant mediators in the association between exposure to misinformation and behavioral intention. 

The Appraisal Tendency Framework distinguishes differences between discrete emotions and cautions grouping emotions into categories such as “negative,” for example with the case of fear and anger, both of which we included in our study [21]. It is important to consider targeting specific emotions when developing interventions or corrective materials to combat misinformation and encourage less risk behavior. For example, prior work using the Appraisal Tendency Framework to understand discrete emotions and smoking found that sadness, but not all negative emotions, heightens addictive behaviors, and posits that messages triggering sadness could unintentionally increase cigarette cravings among smokers [25]. Although sadness was not a discrete emotion in our study, this theory and prior research supports why certain emotions in our study were significant but not others. This could explain why in some pathways “worried” was significant, but “scared” was not.

Our findings will inform future intervention design to target corrective information that addresses specific emotions. For instance, smokers were less likely to purchase e-cigarettes after viewing tweets that e-cigarettes are just as harmful as or more harmful than smoking (relative to those who viewed control tweets) and this difference was partly mediated by reporting they were worried after reading the tweet, potentially preventing people who smoke from reducing their harm if they were to switch to e-cigarettes completely [12]. This would suggest that an intervention to mitigate the effect of such misinformation on e-cigarette purchase intentions would also need to consider information that allay smokers’ worry about the relative harms of e-cigarettes compared with smoking.

We further learnt that while certain emotions and perceived relative harms separately mediated the effects of exposure to conditions on intentions to purchase, there was evidence to support that certain emotions were serial mediators of these effects through perceived relative harms. This finding provides preliminary evidence to suggest that emotions and cognitions are integrally connected in the pathway of misinformation processing and acceptance and that future research should include both affective and cognitive measures when examining misinformation effects on population behaviors. We found support for the serial mediation hypotheses for 4 of the 6 emotions (scared, worried, angry, and hopeful). Future research should further explore the underlying reasons why the activation of these emotions was uniquely associated with processing of e-cigarette harms and potential ways to leverage this evidence in intervention design.

This study has a few limitations. First, we measured self-reported emotional responses, perceived relative harm, and intention to purchase e-cigarettes. Future work could consider measuring psychophysiological responses, such as skin conductance or heart rate, as measures of emotional arousal to misinformation. Second, we are unable to determine the causal direction between the self-reported measures. For instance, perceived relative harm may conceivably influence one’s emotional response. We further conducted sensitivity analyses reversing the order of perceived relative harm and emotions and this model also had an acceptable model fit. Further research to examine the causal relationships between emotional response and perceived relative harm in the context of misinformation will be needed. The study involved a brief exposure of misinformation stimuli in a single sitting and within an online survey. We were therefore not able to determine effects of repeated exposure in the more naturalistic setting of viewing misinformation within participants’ social media accounts. Future studies could consider replicating this research by measuring the impact of e-cigarette misinformation exposure when participants viewed such information during routine use of social media. Neither did we examine whether the mediating pathways would differ across population subgroups. It is possible that specific emotions and perceived relative harms may be differentially activated and mediate the exposure–intention relationship between populations based on their vulnerability to misinformation (e.g., conspiracy ideation, health literacy, or access to accurate health information) [13]. Future research is needed to identify those who are more susceptible to misinformation effects and to examine if the study findings would be replicated in these populations.

## 5. Conclusions

In sum, these findings suggest the importance of considering both affective and psychological responses of health misinformation on social media as mediating mechanisms when assessing the effect of such misinformation among adult smokers. In cases such as exposure to misinformation, it may be easier to intervene on a mediator rather than the exposure to address behavioral intentions to purchase e-cigarettes [26], especially since it is difficult to regulate the online content people are regularly exposed to. Education and corrective messaging that incorporate specific emotions and address perceived relative harms may reduce the negative impacts of exposure to misinformation of e-cigarettes. These study findings will inform further theorizing of the mechanisms of misinformation effects on behavioral intentions and potential approaches to mitigate adverse effects of exposure to misinformation on social media.

## Figures and Tables

**Table 1:** Socio-demographic characteristics of study sample by experimental condition and country.

	US				UK			
	Condition 1	Condition 2	Condition 3	Condition 4	Condition 1	Condition 2	Condition 3	Condition 4
Characteristics	n = 300	n = 300	n = 300	n = 300	n = 300	n = 300	n = 300	n = 300
Age: Mean (SD)								
	50.5 (13.6)	50.0 (13.6)	50.0 (14.7)	50.3 (13.5)	44.1 (14.6)	44.2 (14.4)	44.0 (14.8)	42.8 (14.6)
Sex: No. (%)								
Female	153 (51.0)	154 (51.3)	154 (51.3)	140 (46.7)	126 (42.0)	136 (45.3)	125 (41.7)	135 (45.0)
US Race: No. (%)								
White	206 (68.7)	214 (71.3)	211 (70.3)	220 (73.3)				
Black or African American	51 (17.0)	47 (15.7)	52 (17.3)	51 (17.0)				
Other ethnicity	43 (14.3)	39 (13.0)	37 (12.3)	29 (9.7)				
UK Ethnicity: No. (%)								
White					284 (94.7)	276 (92.0)	278 (92.7)	282 (94.0)
Other ethnicity					16 (5.3)	24 (8.0)	22 (7.3)	18 (6.0)
Education: No (%)								
High/Secondary school or below	83 (27.7)	99 (33.0)	91 (30.3)	89 (29.7)	118 (39.3)	126 (42.0)	122 (40.7)	129 (43.0)
Some college/further education college	111 (37.0)	122 (40.7)	123 (41.0)	110 (36.7)	110 (36.7)	103 (34.3)	105 (35.0)	105 (35.0)
College/University degree or higher	106 (35.3)	79 (26.3)	86 (28.7)	101 (33.7)	72 (24.0)	71 (23.7)	73 (24.3)	66 (22.0)
Smoking status: Mean (SD)								
No. days smoked in last 30 days	28.9 (4.2)	27.8 (5.9)	27.7 (5.9)	28.1 (5.4)	27.5 (6.3)	27.4 (6.9)	26.7 (7.7)	27.1 (7.00)
E-cigarette use: No (%)								
Never used e-cigarettes	145 (48.3)	144 (48.0)	152 (50.7)	158 (52.7)	138 (46.0)	124 (41.3)	152 (50.7)	148 (49.3)
Social Media use: Mean (SD)								
Range 0–8	1.62 (1.46)	1.76 (1.49)	1.80 (1.63)	1.72 (1.56)	2.11 (1.65)	2.24 (1.71)	2.19 (1.77)	2.19 (1.64)
Daily Internet use: Mean (SD)								
Range 0–24	6.59 (4.77)	6.89 (4.72)	6.24 (4.37)	6.48 (4.37)	5.18 (3.70)	5.54 (4.01)	5.80 (3.80)	5.65 (3.92)

Notes. Test for variance across conditions; continuous variables analyzed using one-way ANOVA test, categorical variables analyzed using χ2 test.

**Table 2:** Descriptive statistics and zero-order correlations between key variables.

	Mean	SD	1	2	3	4	5	6	7	8	9	10
1. Baseline intention to purchase e-cigarette	1.480	2.321	1	**0.844**	−**0.251**	−**0.231**	**0.057**	0.023	−0.008	**0.276**	**0.336**	**0.274**
2. Post-test intention to purchase e-cigarette	1.390	2.347	**0.844**	1	−**0.236**	**−0.276**	0.019	−0.024	−0.037	**0.323**	**0.377**	**0.308**
3. Baseline relative harm ^a^	3.175	1.280	−**0.251**	−**0.236**	1	**0.727**	**0.127**	**0.115**	**0.126**	−0.032	−0.022	−0.005
4. Post-test relative harm ^b^	3.198	1.279	−**0.231**	−**0.276**	**0.727**	1	**0.207**	**0.198**	**0.213**	−**0.70**	−**0.044**	−0.018
5. Scared	2.070	1.253	**0.056**	0.019	**0.127**	**0.207**	1	**0.779**	**0.571**	**0.133**	**0.069**	**0.149**
6. Worried	2.330	1.294	0.023	−0.024	**0.115**	**0.198**	**0.779**	1	**0.574**	**0.107**	0.028	**0.096**
7. Angry	1.950	1.224	−0.008	−0.037	**0.126**	**0.213**	**0.571**	**0.574**	1	0.021	0.012	**0.088**
8. Hopeful	1.828	1.091	**0.276**	**0.323**	−0.032	−**0.070**	**0.133**	**0.107**	0.021	1	**0.674**	**0.689**
9. Happy	1.560	0.951	**0.336**	**0.377**	−0.022	−**0.043**	**0.069**	0.028	0.012	**0.674**	1	**0.687**
10. Relieved	1.621	1.000	**0.274**	**0.308**	−0.005	−0.018	**0.149**	**0.096**	**0.088**	**0.689**	**0.687**	1

Note. ^a^ Missing values among 177 participants. ^b^ Missing values among 169 participants. Pairwise Pearson’s correlation coefficients (r) are presented. All bolded results are significant at *p* < 0.05.

**Table 3:** Mean (standard deviation) of emotions, baseline and post-test perceptions of relative harm of e-cigarettes, and baseline and post-test intention to purchase e-cigarettes, by condition.

	Emotions						Baseline Perceptions of Relative Harm	Post-Test Perceptions of Relative Harm	Baseline Intention to Purchase e-Cigarettes	Post-Test Intention to Purchase e-Cigarettes
	Scared	Hopeful	Worried	Happy	Angry	Relieved				
All										
Condition 1	2.44 (1.33)	1.61 (1.04)	2.71 (1.33)	1.32 (0.78)	2.45 (1.34)	1.53 (1.00)	3.12 (1.27)	3.37 (1.19)	1.50 (2.31)	1.09 (2.09)
Condition 2	1.92 (1.21)	1.70 (1.00)	2.15 (1.30)	1.49 (0.91)	1.90 (1.21)	1.58 (0.92)	3.24 (1.32)	3.10 (1.34)	1.35 (2.21)	1.49 (2.43)
Condition 3	2.11 (1.22)	1.86 (1.07)	2.44 (1.25)	1.58 (0.96)	2.11 (1.22)	1.61 (0.99)	3.12 (1.25)	3.16 (1.30)	1.57 (2.39)	1.45 (2.36)
Condition 4	1.83 (1.14)	2.15 (1.18)	2.02 (1.19)	1.84 (1.06)	1.83 (1.14)	1.77 (1.07)	3.21 (1.28)	3.16 (1.27)	1.50 (2.36)	1.53 (2.47)
US										
Condition 1	2.55 (1.42)	1.74 (1.17)	2.82 (1.42)	1.33 (0.84)	2.55 (1.42)	1.56 (1.06)	3.17 (1.03)	3.45 (1.06)	1.33 (2.24)	0.98 (2.02)
Condition 2	2.13 (1.35)	1.64 (1.03)	2.31 (1.41)	1.45 (0.90)	2.13 (1.35)	1.55 (0.95)	3.35 (1.28)	3.15 (1.12)	1.15 (2.08)	1.30 (2.27)
Condition 3	2.28 (1.32)	1.81 (1.10)	2.60 (1.32)	1.49 (0.93)	2.28 (1.32)	1.53 (0.93)	3.20 (1.04)	3.22 (1.03)	1.25 (2.20)	1.16 (2.17)
Condition 4	2.01 (1.29)	2.19 (1.20)	2.16 (1.29)	1.87 (1.12)	2.01 (1.29)	1.81 (1.14)	3.26 (1.10)	3.22 (1.07)	1.29 (2.23)	1.27 (2.31)
UK										
Condition 1	2.34 (1.24)	2.19 (1.20)	2.60 (1.22)	1.31 (0.70)	2.34 (1.24)	1.49 (0.93)	2.64 (0.95)	3.02 (1.00)	1.67 (2.37)	1.21 (2.16)
Condition 2	1.68 (1.01)	1.48 (0.88)	1.98 (1.16)	1.54 (0.93)	1.68 (1.01)	1.61 (0.90)	2.67 (0.93)	2.60 (0.98)	1.57 (2.33)	1.68 (2.56)
Condition 3	1.94 (1.10)	1.76 (0.96)	2.28 (1.15)	1.68 (0.99)	1.94 (1.10)	1.69 (1.04)	2.60 (0.90)	2.60 (0.93)	1.88 (2.54)	1.73 (2.50)
Condition 4	1.65 (0.94)	1.90 (1.04)	1.88 (1.05)	1.81 (0.99)	1.64 (0.94)	1.73 (1.00)	2.68 (0.90)	2.66 (0.92)	1.71 (2.47)	1.79 (2.61)

**Table 4:** Total, direct, and indirect effects for mediation analyses.

	Effect Size	Bias-Corrected Bootstrap 95% CI	
**Total Effects (Direct + Indirect Effects)**			
Condition 1 → Intention to Purchase E-cigarettes	**−0.483**	**−0.633**	**−0.334**
Condition 2 → Intention to Purchase E-cigarettes	0.083	−0.063	0.216
Condition 3 → Intention to Purchase E-cigarettes	−0.138	−0.279	0.008
**Direct Effects**			
Condition 1 → Intention to Purchase E-cigarettes	**−0.288**	**−0.437**	**−0.135**
Condition 2 → Intention to Purchase E-cigarettes	**0.145**	**0.002**	**0.280**
Condition 3 → Intention to Purchase E-cigarettes	−0.063	−0.201	0.079
**H1: Indirect Effects through Relative Harm:** Condition → Relative Harm → Intention to Purchase E-cigarettes			
Condition 1 → Relative Harm → Intention to Purchase E-cigarettes	**−0.048**	**−0.072**	**−0.030**
Condition 2 → Relative Harm → Intention to Purchase E-cigarettes	**0.032**	**0.018**	**0.051**
Condition 3 → Relative Harm → Intention to Purchase E-cigarettes	0.010	−0.003	0.023
**H2: Indirect Effects through Emotion:** Condition → Emotion → Intention to Purchase E-cigarettes			
Condition 1 → Scared → Intention to Purchase E-cigarettes	0.035	−0.010	0.088
Condition 2 → Scared → Intention to Purchase E-cigarettes	0.004	−0.002	0.021
Condition 3 → Scared → Intention to Purchase E-cigarettes	0.016	−0.004	0.044
Condition 1 → Worried → Intention to Purchase E-cigarettes	**−0.053**	**−0.107**	**−0.009**
Condition 2 → Worried → Intention to Purchase E-cigarettes	−0.008	−0.030	0.000
Condition 3 → Worried → Intention to Purchase E-cigarettes	**−0.029**	**−0.062**	**−0.006**
Condition 1 → Angry → Intention to Purchase E-cigarettes	0.035	−0.011	0.090
Condition 2 → Angry → Intention to Purchase E-cigarettes	0.016	−0.005	0.040
Condition 3 → Angry → Intention to Purchase E-cigarettes	0.019	−0.005	0.049
Condition 1 → Hopeful → Intention to Purchase E-cigarettes	**−0.052**	**−0.101**	**−0.012**
Condition 2 → Hopeful → Intention to Purchase E-cigarettes	**−0.042**	**−0.085**	**−0.011**
Condition 3 → Hopeful → Intention to Purchase E-cigarettes	**−0.030**	**−0.064**	**−0.007**
Condition 1 → Happy → Intention to Purchase E-cigarettes	**−0.075**	**−0.134**	**−0.027**
Condition 2 → Happy → Intention to Purchase E-cigarettes	**−0.046**	**−0.088**	**−0.018**
Condition 3 → Happy → Intention to Purchase E-cigarettes	**−0.039**	**−0.079**	**−0.014**
Condition 1 → Relieved → Intention to Purchase E-cigarettes	−0.012	−0.038	0.007
Condition 2 → Relieved → Intention to Purchase E-cigarettes	−0.008	−0.028	0.003
Condition 3 → Relieved → Intention to Purchase E-cigarettes	−0.009	−0.031	0.004
**H3: Indirect Effects through Emotion and Relative Harm: ** Condition → Emotion → Relative Harm → Intention to Purchase E-cigarettes			
Condition 1 → Scared → Relative Harm → Intention to Purchase E-cigarettes	**−0.007**	**−0.015**	**−0.002**
Condition 2 → Scared → Relative Harm → Intention to Purchase E-cigarettes	−0.001	−0.003	0.001
Condition 3 → Scared → Relative Harm → Intention to Purchase E-cigarettes	**−0.003**	**−0.008**	**−0.001**
Condition 1 → Worried → Relative Harm → Intention to Purchase E-cigarettes	−0.006	−0.013	0.000
Condition 2 → Worried → Relative Harm → Intention to Purchase E-cigarettes	−0.001	−0.004	0.000
Condition 3 → Worried → Relative Harm → Intention to Purchase E-cigarettes	**−0.003**	**−0.008**	**−0.0001**
Condition 1 → Angry → Relative Harm → Intention to Purchase E-cigarettes	**−0.009**	**−0.017**	**−0.003**
Condition 2 → Angry → Relative Harm → Intention to Purchase E-cigarettes	**−0.004**	**−0.008**	**−0.002**
Condition 3 → Angry → Relative Harm → Intention to Purchase E-cigarettes	**−0.005**	**−0.010**	**−0.002**
Condition 1 → Hopeful → Relative Harm → Intention to Purchase E-cigarettes	**−0.007**	**−0.013**	**−0.002**
Condition 2 → Hopeful → Relative Harm → Intention to Purchase E-cigarettes	**−0.005**	**−0.010**	**−0.002**
Condition 3 → Hopeful → Relative Harm → Intention to Purchase E-cigarettes	**−0.004**	**−0.009**	**−0.001**
Condition 1 → Happy → Relative Harm → Intention to Purchase E-cigarettes	0.002	−0.003	0.007
Condition 2 → Happy → Relative Harm → Intention to Purchase E-cigarettes	0.001	−0.002	0.004
Condition 3 → Happy → Relative Harm → Intention to Purchase E-cigarettes	0.001	−0.001	0.004
Condition 1 → Relieved → Relative Harm → Intention to Purchase E-cigarettes	0.001	−0.001	0.004
Condition 2 → Relieved → Relative Harm → Intention to Purchase E-cigarettes	0.001	−0.001	0.003
Condition 3 → Relieved → Relative Harm → Intention to Purchase E-cigarettes	0.001	−0.001	0.003

Note. The referent condition is Condition 4 (physical activity control tweets). N = 2167 (233 cases were missing either baseline or post-test perceived relative harm). All bolded effects are nonzero. These analyses report the effects of the paths from the first named variable to the last named variable through the mediator, adjusting for baseline intention to purchase e-cigarettes and perceived relative harm of e-cigarettes. The bootstrap procedure was used to compute the bias-corrected 95% confidence intervals.

## Data Availability

Data are available upon reasonable request. The data are deidentified participant data as outlined at http://www.isrctn.com/ISRCTN16082420 (28 October 2021). The data will be made available upon reasonable requests. We also plan to publish the data from the study once all research outlined in our research proposal has been submitted for publication.

## Supplementary Materials

The following are available online at https://www.mdpi.com/article/10.3390/ijerph182312347/s1, Supplementary File: Message stimuli with each condition.
