# Supplementary material for: Emotional Responses and Perceived Relative Harm Mediate the Effect of Exposure to Misinformation about E-Cigarettes on Twitter and Intention to Purchase E-Cigarettes among Adult Smokers
Source: Int J Environ Res Public Health. 2021 Nov 24;18(23):12347. doi: 10.3390/ijerph182312347 (PMC8656833; doi:10.3390/ijerph182312347)
Supplement: Supplementary file 1 [file ijerph-18-12347-s001.zip › ijerph-1431334-supplementary.pdf]

Supplementary Material

Message stimuli with each condition

Condition 1: E-cigarette is more harmful

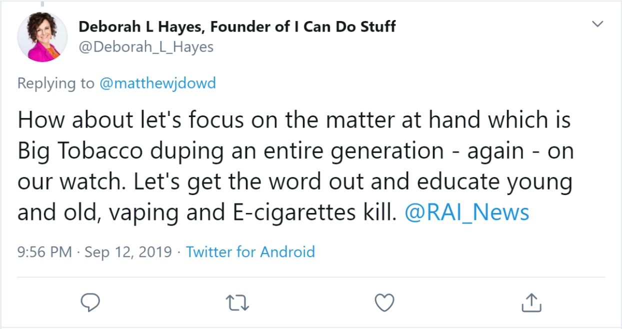

Deborah L Hayes, Founder of I Can Do Stuff
@Deborah_L_Hayes

Replying to @matthewjdowd

How about let's focus on the matter at hand which is Big Tobacco duping an entire generation - again - on our watch. Let's get the word out and educate young and old, vaping and E-cigarettes kill. @RAI_News

9:56 PM · Sep 12, 2019 · Twitter for Android

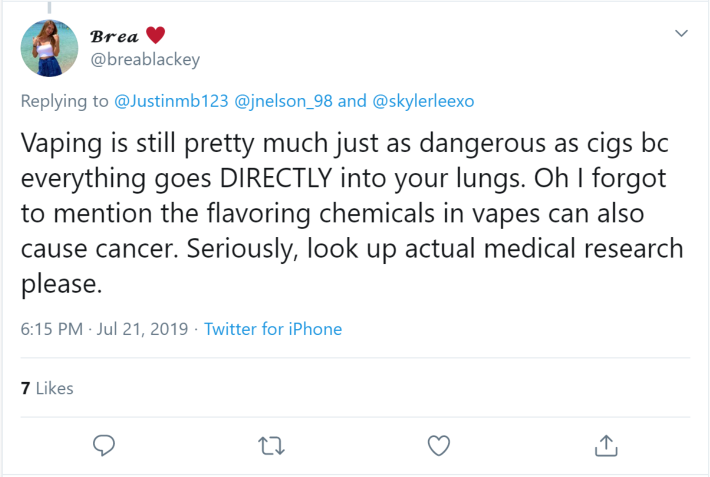

Brea ❤
@breablackey

Replying to @Justinmb123 @jnelson_98 and @skylerleexo

Vaping is still pretty much just as dangerous as cigs bc everything goes DIRECTLY into your lungs. Oh I forgot to mention the flavoring chemicals in vapes can also cause cancer. Seriously, look up actual medical research please.

6:15 PM · Jul 21, 2019 · Twitter for iPhone

7 Likes

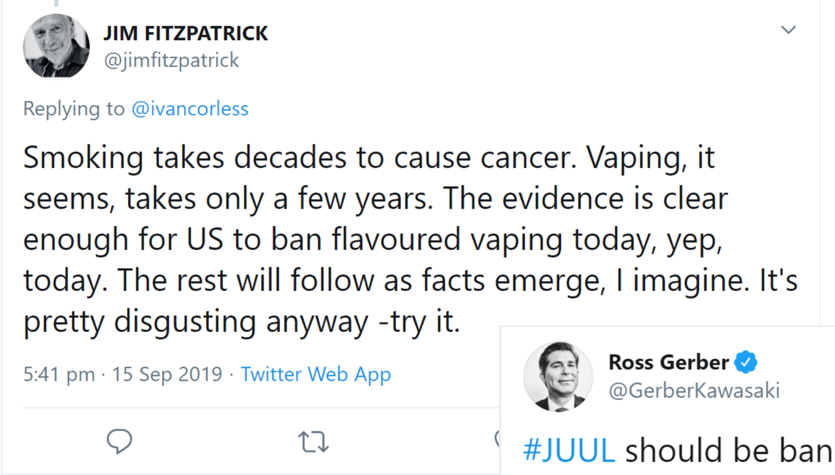

JIM FITZPATRICK
@jimfitzpatrick

Replying to @ivancorless

Smoking takes decades to cause cancer. Vaping, it seems, takes only a few years. The evidence is clear enough for US to ban flavoured vaping today, yep, today. The rest will follow as facts emerge, I imagine. It's pretty disgusting anyway -try it.

5:41 pm · 15 Sep 2019 · Twitter Web App

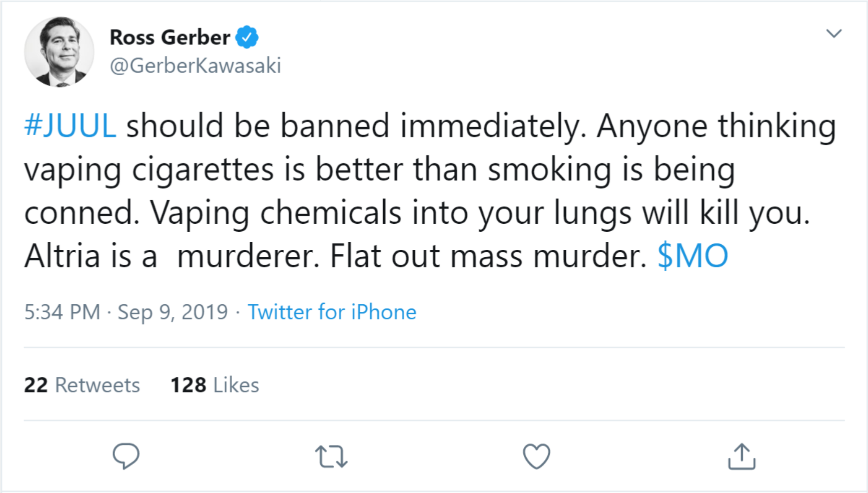

Ross Gerber
@GerberKawasaki

#JUUL should be banned immediately. Anyone thinking vaping cigarettes is better than smoking is being conned. Vaping chemicals into your lungs will kill you. Altria is a murderer. Flat out mass murder. $MO

5:34 PM · Sep 9, 2019 · Twitter for iPhone

22 Retweets 128 Likes

Condition 2: E-cigarette is completely harmless

Clint Plumingtons (Glause Black) 🤘🖖✌️🗣️💨🙊🙉🙈
@DarthMods

Replying to @d3_gardner

WOW. You're a doctor and you are spreading this fearmonger propaganda? What happened to your oath to do no harm?

There are ZERO proven harms in the 15 years vaping has existed when used in the suggested parameters.

I highly suggest you educate yourself on all the facts.. 1/2

7:47 AM · Jul 16, 2019 · Twitter Web Client

G. Karl Snæ MD ✤
@KalliSnae

Replying to @ShereefElnahal

Oh, it's not only safer, they are SAFE - or, you know of any harm by vaping tho ~15y on the market and ~50.000.000 users world-wide? - and ~8.000 flavours! No, didn't think so bcos NONE so far - NONE - that'ts how SAFE vaping is - did say vaping. Any objections to that?

3:40 AM · May 25, 2019 from Iceland · Twitter for iPhone

5 Retweets **15** Likes

❄️**Got Janie?** 🌊🐢💨🍄
@GotJanie

Replying to @marycaddell

I'm an asthmatic lol. I know the science behind vaping. It's completely safe. Big tobacco scares ppl. Like thruth dot .org... big tobacco supports them. It's crazy.

5:13 PM · Jul 4, 2019 · Twitter for iPhone

DyNama
@DyNama

I don't worry about the ingreds of ejuice for #vaping, they are harmless, but I do wonder about the artificial breathing, the regular deep puffing. Do trumpet players get a breathing disorder? My puffing #ecigs is kind of like that.

6:21 PM · Jul 15, 2019 · TweetCaster for Android

Condition 3: Uncertainty

André Picard
@picardonhealth

We Still Don't Know How Safe #Vaping Is - it's time to get more information about the risks of #ecigarettes: @nytimes editorial

12:13 PM · Sep 6, 2019 · Twitter Web Client

7 Retweets **15** Likes

Mom Folding Laundry
@JenandZen

This whole anti-vaping schtick is cooked up by drug regulation & enforcement to make sure the MONEY keeps flowing to their coffers.

I have yet to see a single credible piece of evidence showing that vaping causes real harm.

(As in more harm than drinking too much coffee.)

7:33 PM · Jul 9, 2019 · Twitter Web App

6 Retweets **32** Likes

ali
@pmoreandahalf

And people are like "but it's not that bad because it's not smoke" ok but nicotine is harmful with or without smoke and there is very limited research done on e-cigs so the FDA doesn't know how harmful they actually are to the extent that we know that cigarettes are harmful

> **RAVEN ELYSE** @RavenElyseTV · Jul 21
> I'm legitimately angry about the fact that the "quit smoking/cigarettes are disgusting" thing was going SO WELL! Smoking was actually going out of style! But then some asshole invented the juul and now a bunch of 12 year olds are addicted to nicotine.

7:36 PM · Jul 21, 2019 · Twitter for iPhone

2 Retweets **13** Likes

NotComey
@notComey

Is San Francisco's vaping ban backed by science?: San Francisco has decided to ban the sale of e-cigarettes in 2020, hoping to curb a surge in vaping among adolescents. But is the policy backed up by the available evidence? – How harmful is vaping? –... rawstory.com/2019/06/is-san...

1:10 AM · Jun 27, 2019 · dlvr.it

2 Retweets

Condition 4: Physical Activity

Jen 🖤 BTS + Fitness
@therealjenlewis

Today reinforces my passion to push the need to exercise for not only the physical benefits. Get out and do something active for your mental health. Go for a walk and clear your mind. Find someone to join you & talk to them. My prayers go out to all today ❤️

3:56 PM · Oct 14, 2019 · Twitter for iPhone

81 Retweets **289** Likes

Sport England
@Sport_England

It's #WorldMentalHealthDay 🎗️ and we know sport and physical activity can have a powerful and positive effect on our mental wellbeing. That's why we invest in projects that are changing lives.

11:46 AM · Oct 10, 2019 · Twitter Ads Composer

84 Retweets **167** Likes

World Health Organization (WHO)
@WHO

Adults (18+) need 150 minutes per week of moderate-intensity physical activity to improve and maintain health.

Let's #BeActive!

bit.ly/2Q3wust

4:57 PM · Sep 7, 2018 · Twitter Web Client

330 Retweets **423** Likes

F.DharanboodhooHC
@dharanboodhoohc

Physical activity & Exercise can have immediate and long-term health benefits. Most importantly, regular activity can improve our quality of life.

7:36 PM · Oct 10, 2019 · Twitter for Android

5 Retweets **6** Likes
